# Patient Preferences for Electronic Versus Paper Patient Information Leaflets: A Survey Among Patients in Sweden

**DOI:** 10.1007/s43441-026-00929-9

**Published:** 2026-02-07

**Authors:** Annika Forsberg, Wenche Olsen Boström, Klaus Kaae Andersen, Daniel Eek

**Affiliations:** 1 AstraZeneca, Medical and Regulatory, Forskaren, Stockholm, Sweden; 2 Omicron, Copenhagen, Denmark

**Keywords:** Patient information leaflet (PIL), Electronic product information, Electronic patient information leaflet (ePIL), Patient preference

## Abstract

**Objectives:**

In compliance with legal requirements, medications are mandated to include a patient information leaflet (PIL) in a physical/paper format that serves as a vital source of information about the medication. While the use of an electronic PIL (ePIL) ensures the availability of the most current information for patients, the prevailing preference between the traditional PIL and ePIL remains uncertain. This observational study aimed to address this gap by reporting the findings of an online survey designed to compare patient preferences for an ePIL versus a paper PIL.

**Methods:**

Conducted across a total of 15 pharmacies in Sweden, the survey enlisted patients via convenience sampling to rate their preference for an ePIL versus a PIL on a 7-point scale together with other questions on, for example, the ease of information retrieval after their visit to the pharmacy. Data collection commenced in December 2022 and ended in November 2023. The primary hypothesis posited that the ePIL format would emerge as the preferred choice. Most participants were female (66%), and the average age was 56 years.

**Results:**

The findings highlighted an inclination towards the ePIL as the preferred choice (58% preferred ePIL, 11% preferred PIL, and 31% were indifferent), with respondents expressing that information retrieval was notably more convenient compared to the paper-based format. Older participants were generally indifferent towards the format. The majority of patients reported familiarity with receiving information in digital formats.

**Conclusions:**

These results suggest the readiness of patients in Sweden to transition to ePILs from the traditional paper PIL.

**Supplementary Information:**

The online version contains supplementary material available at 10.1007/s43441-026-00929-9.

## Introduction

In compliance with Swedish legislation, marketing authorisation holders must include a physical/paper Patient Information Leaflet (PIL) for every pharmaceutical package, to be provided to a patient upon its dispensing [1, 2]. The Swedish Medical Products Agency (MPA) currently views electronic Product Information (ePI) as a supplement to the paper PIL. ePI is a semi-structured digital format that encompasses structured elements (fixed headings, controlled vocabularies) and unstructured elements (free text, graphics), and includes the Summary of Product Characteristics (SmPC), the PIL, and labelling [3]. The European Commission mandates a package leaflet except where all required information can be conveyed on the labelling [4]. In practice, the electronic Patient Information Leaflet (ePIL) refers to the electronic version of the package leaflet.

The PIL is based on the content of the SmPC, which is primarily tailored for healthcare providers, delivers vital information about medications to end-users, adhering to a standard template for all medications, and provides patients with essential information on medicine use, risks, and safe administration [5, 6]. Historically, PILs evolved from early persuasive pamphlets to regulated patient-education tools, reflecting broader shifts toward patient-centred care and transparency. This evolution is documented in corpus-assisted analyses of PILs which show a move from promotional rhetoric to standardized, informational content aligned with regulatory standards and patient autonomy [7]. In the EU, PILs must be clear, legible, and easy to use in the local language and they are updated following changes in the SmPCs or as required by health authorities [5]. When a new PIL is approved by health authorities, the leaflet inside packs on the market becomes outdated. In Sweden, an updated PIL must be implemented within six months of approval, after which packs with the old leaflet cannot be released to the market.

ePILs offer several potential advantages: timely implementation of updates, improved accessibility features (zoom, text-to-speech), machine readability, and the ability to link to multimedia instructions and national health portals, in addition to environmental benefits. Transitioning away from paper could yield substantial sustainability gains. For instance, a large pharmaceutical company, such as AstraZeneca, could potentially save 500,000 trees, 50,000 eCO_2_ (equivalent carbon dioxide), and 1.6 billion litres of water annually by eliminating paper leaflets in supplied packs globally (excluding ink and transport of printed leaflets) (Data on File), consistent with broader digital ambitions in EU medicines information [6, 8, 9]. In 2017, the European Commission underscored the need to enhance the leaflet and SmPC using digital tools to better serve patients and healthcare professionals [6]. However, regulatory modernisation also requires careful attention to equity, readability, and user-testing safeguards. Recent analyses highlight that current EU mandatory user-testing policies for PILs face challenges in diversity and independence of testing samples, and limited protocols for translated versions, potentially affecting comprehensibility across patient groups [10].

Sweden presents a favourable context for digital transition: in 2023, 96% of Swedes aged 16–85 used the internet within the last three months [11]. In a survey conducted in 2023, one-third of Swedes ordered prescribed medicines online from licensed pharmacies in the previous two years [12]. These patterns suggest high readiness overall, with identifiable groups potentially needing tailored support.

The present survey aimed to compare patient perspectives on using ePILs versus paper PILs in Sweden and to test the hypothesis that patients prefer ePILs. Beyond preference, we explicitly focus on constructs related to information access and retrieval (“findability”), while acknowledging that comprehension (i.e., accurate interpretation and application of information) is also distinct but was beyond the scope of our study. Prior work shows that even when sections are easy to locate, benefit information is often under-specified in PILs and rarely quantified, which affects informed decision-making [13]. Our objectives were to assess preference, perceived ease of finding information across formats, readiness for digital tools, and potential obstacles to transitioning from paper PILs to ePILs, with attention to demographic differences that could inform supportive implementation strategies.

## Materials and Methods

### Study Setting and Survey

This was an observational survey study on ePIL usage for which patients were recruited via convenience sampling through a collaboration with the pharmacy chain Apoteket AB in a total of 15 pharmacies in Sweden.

Pharmacists underwent training by the project lead at the pharmacy chain. All patients visiting the pharmacies during the data collection period (December 2022–November 2023) that were prescribed AstraZeneca Turbuhaler products were considered eligible for study participation. They were invited to participate when collecting the prescribed product, ensuring a diverse sample due to the wide age range of users.

For patients that declined participation, recruiting pharmacists received a questionnaire link to document reasons for non-participation, including whether the product was collected for someone else (yes/no), the patient’s gender and age, and the primary reason for not participating in the survey.

Patients who consented to participate received written information about the study from the pharmacists to bring back home for their later consideration, including a link to the online survey. Those collecting product(s) for someone else were also eligible to participate. To access the ePIL, the well-known and frequently used FASS (“Farmacevtiska specialiteter i Sverige”) website and its associated app served as the primary sources. Patients with parallel-imported products were excluded, as the ePIL could only be published on FASS with a prior agreement from the pharmaceutical company. The pharmaceutical packs used in the survey were standard market packs containing paper PILs. The survey included preference questions instructing participants to review both the paper PIL (included in the package) and the ePIL. For the ePIL, participants could choose to either search the FASS website or use the FASS app to scan the 2D-barcode on the carton. Then they compared the two formats by responding to ten survey questions.

The study-specific online survey comprised background questions on the patient’s gender, age, and duration of product use (first-time user/less than a year/1–3 years/4–6 years/more than 6 years), as well as six questions that used 7-point rating scales on factors including the patients’ frequency of reading the PIL, preference for paper PIL or ePIL, ease of finding the ePIL, ease of finding information in the ePIL, ease of finding information in the PIL, and the frequency of digital information searches in general. The complete survey is included in Supplementary Materials.

### Statistical Analysis

The aim of this study was to demonstrate a preference for the ePIL over the paper PIL. The primary endpoint was the rating of Question 2: “If it was possible to choose, would you prefer to receive the leaflet only in paper or only in digital format?” (1 = only paper, 4 = format doesn’t matter, 7 = only digital). The null hypothesis of no preference was H0: μ = 4, where μ was the mean rating of Question 2.

Prior to data collection, a sample size of 72 respondents was considered sufficient to test the hypothesis, calculated to detect a difference of at least 1 point above 4 on the 7-point response scale in favour of the ePIL with 80% power at a 5% significance level. To account for potential dropouts and incomplete data, the goal was to recruit up to 100 respondents.

All analyses were pre-specified in a statistical analysis plan. The primary hypothesis was tested at a 5% significance level using a t-test. A one-sample Wilcoxon signed-rank test was used as a sensitivity analysis, being robust to outliers and not assuming normality. Furthermore, to address potential bias from non-participation, a sensitivity analysis was performed on the primary endpoint, including participant characteristics (gender and age) as weights to balance differences between respondents and non-respondents.

Several exploratory analyses were conducted to determine if the preference for ePIL could be explained by patient characteristics or other factors measured by the survey questions. This was done by regressing the preference for ePIL on patient characteristics and survey questions, with associations quantified using Spearman correlations. No adjustments for multiple comparisons were applied due to the exploratory nature of these analyses.

All results are presented descriptively, including tables and figures, with estimates provided alongside 95% Confidence Intervals (CIs).

## Results

### Participant Demographics

Recruitment for the survey commenced on 2 December 2022 and concluded for recruiting pharmacies on 15 November 2023, but remained open for patient completion until 20 November 2023 to allow sufficient time for responses. In total, 857 patients visited the pharmacies to collect their AstraZeneca Turbuhaler products during the study period and were invited by the recruiting pharmacists to participate of whom 249 (29%) declined participation and 608 (71%) consented to bring the study information back home for their later consideration. Of the latter, 103 (17% [103/608]) participated in the study by completing the online questionnaire resulting in an overall response rate of 12% (103/857).

The primary reason for non-participation among the 249 patients that declined participation was a lack of time (47%). Table 1 presents a comparison of the demographics of respondents and non-respondents. An external analysis revealed no significant difference in gender distribution between the two groups (*p* > 0.5); however, non-respondents were generally older than respondents (*p* < 0.05).

**Table 1:** Demographics for respondents and non-respondents

Variable	Respondents (*n* = 103)	Non-respondents (*n* = 249)
*Age group, N (%)*		
15–30	5 (5)	15 (6)
31–50	21 (20)	60 (24)
51–70	52 (57)	104 (42)
70+	14 (15)	68 (27)
Missing	9 (8)	2 (1)
*Gender*		
Female, *n* (%)	68 (66)	154 (62)
Male, *n* (%)	35 (34)	95 (38)

The mean age of the 103 respondents was 56 years, with the majority (66%) having used the treatment for more than 6 years (6% were first-time users, 4% had used the treatment for less than a year, 12% for 1–3 years, and 13% for 4–6 years).

### Survey Responses and Preference Scores

**Fig. 1:**
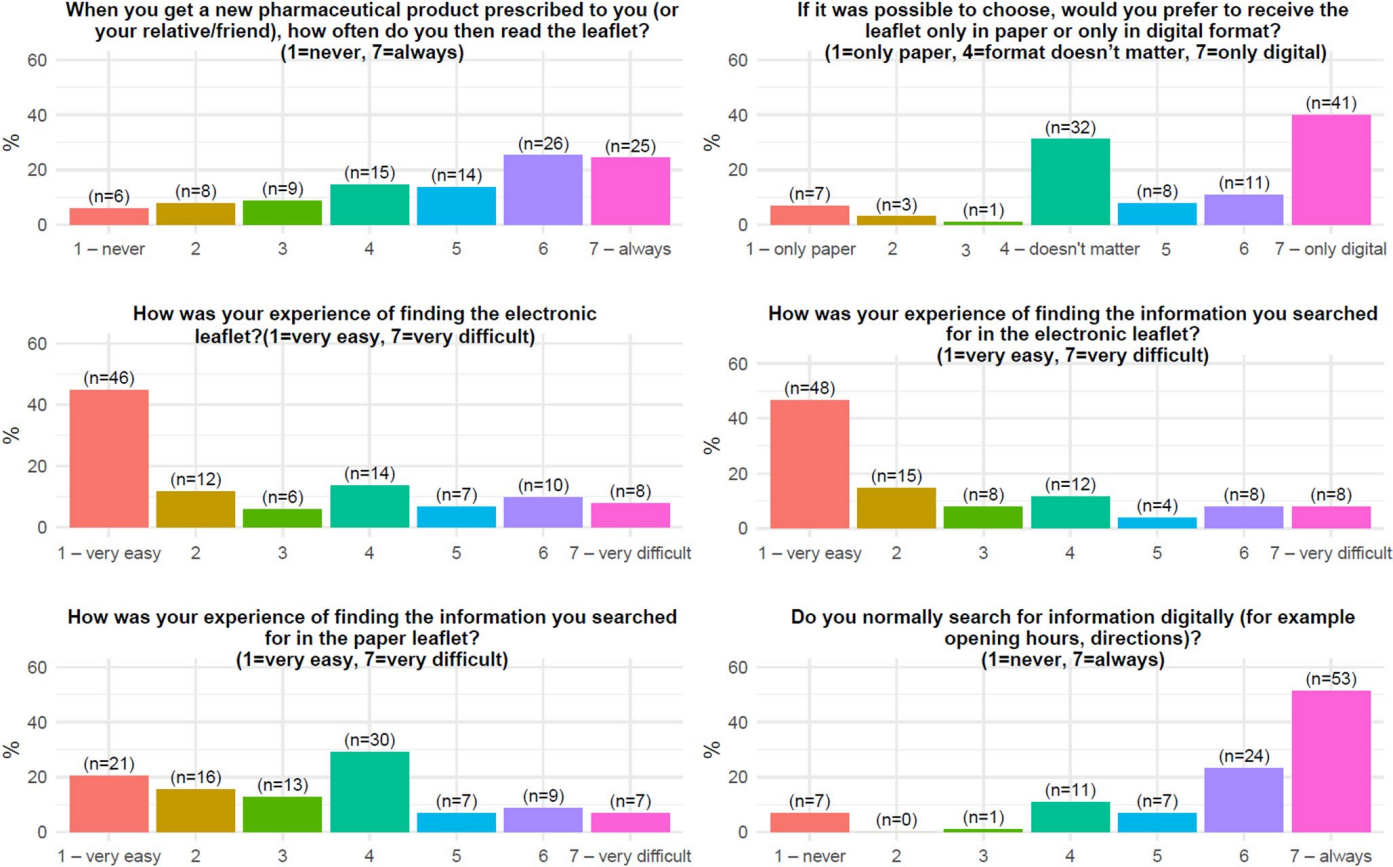
Distribution of responses to survey questions. The y-axis shows proportions for each response category, calculated as the number of responses in each category divided by number of responses

Descriptive results for the survey questions, including the frequency of reading the PIL, preference for leaflet format, and ease of finding information, were analysed. Figure 1 illustrates the distribution of responses to the survey questions, indicating that a large number of patients (63% responding above the mid on the 7-point scale and 22% below) normally read the leaflet when collecting their medication. The majority reported finding it easy to access and retrieve information using both the ePIL and the paper PIL, with more positive scores for the ePIL (47% found the information very easily in the ePIL compared to 20% in the PIL). Moreover, a substantial number of patients expressed familiarity with digital information.

The survey confirmed the primary hypothesis, revealing a statistically significant preference for the ePIL over the paper PIL (*p* < 0.0001). There was no missing data for the primary endpoint. The mean score of the primary endpoint (Question 2) was 5.21 (95% CI 4.8–5.6), indicating a clear preference for the ePIL. Median was 6 (95% CI by bootstrap 4–6) and IQR was 4–7, respectively. The non-parametric test confirmed this significant preference (Wilcoxon-test *p* < 0.0001), and after adjusting the analysis for non-response, the statistically significant preference persisted (*p* < 0.0001). The weighted mean score (weighted to the non-responders) was 4.9 (95% CI 4.4–5.4), confirming the results.

**Fig. 2:**
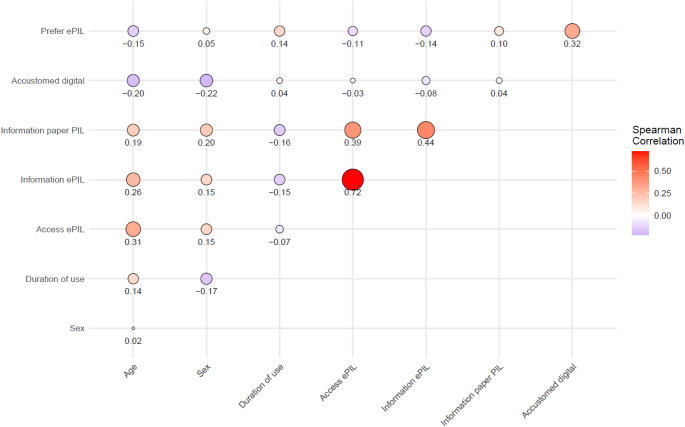
Correlations (Spearman) between survey questions. ePIL, an electronic PIL; PIL, patient information leaflet

The survey data were further analysed using separate linear regression models to explore the correlation of gender, duration of medication use, age, and responses to the survey questions with the preference for the ePIL. Spearman correlations are presented in Fig. 2. Two significant models emerged: one associating the preference for the ePIL with age and the other with the duration of use. As shown in Table 2, preference for the ePIL varied with age; the oldest patient group preferred PIL to ePIL, while all other age groups preferred the ePIL to the PIL. Furthermore, the second significant model showed that patients’ familiarity with searching for digital information increased their preference for the ePIL.

**Table 2:** Preference score for the electronic patient information leaflet by birth year

	Before 1950	1950–1959	1960–1969	1970–1979	1980–1989	After 1990
N	11	23	26	20	7	7
Mean (SD)	3.6 (2.1)	5.3 (1.6)	5.7 (1.5)	4.6 (2.4)	6.3 (1.1)	5.6 (1.3)
Median	4	5	6	4	7	6
Min–max	1–7	2–7	2–7	1–7	4–7	4–7

## Discussion

This survey examined patient preference and perceived ease of locating information in ePIL versus paper PIL formats among users of chronic inhalation therapy in Sweden. We found a statistically significant preference for ePILs overall and indications that patients reported easier access and retrieval of information in the ePIL compared to in the PIL. Age moderated preference since patients born before 1950 showed greater inclination towards PIL or indifference, while most other age groups preferred ePILs. Familiarity with searching for digital information was positively associated with preferring ePILs, consistent with Sweden’s high digital uptake.

Our results address patient preference and perceived ease of locating information. We did not directly assess comprehension, that is the accuracy and speed with which readers interpret specific items (e.g., contraindications, dosing) and apply them to decisions or actions. This distinction matters for regulatory modernisation. EU user-testing frameworks aim to ensure PILs are legible, clear, and easy to use, yet recent analyses point to gaps in testing diversity, independence, and handling of translations, all of which can affect comprehension across populations [10]. In addition, content quality influences comprehension. A content analysis of EU PILs found that benefit information is often under-specified, frequently lacking quantitative data, despite detailed risk frequency descriptors [13]. Thus, even if ePILs improve findability, comprehension and decision alignment depend on what is written (e.g., reason for treatment, duration, whether preventive or symptomatic, and numerical benefit information), not only how content is accessed. Strengthening content (benefit clarity, quantitative outcomes where appropriate) and user-testing safeguards is integral to a safe, equitable digital transition.

Historically, PILs have shifted from persuasive marketing genres to regulated patient-facing materials tied to patient autonomy and safety [7]. Corpus-assisted analyses indicate contemporary PILs emphasise standardized informational content, regulatory compliance, and patient education [9]. Our preference signal for ePIL aligns with the EU’s intent to use digital tools to update and enhance medicines information [6, 7]. The Swedish environment, with widespread internet access and established national portals (e.g., 1177.se), provides infrastructure for ePIL access and integration. Still, modernisation should be anchored in equity, ensuring that patients who prefer paper, lack equipment, or face digital discomfort can access printed PILs, and ensuring translations and accessibility features meet diverse literacy and language needs.

Paper PILs are hard to read, have long implementation lead time, and are burdensome for supply chains and the environment, motivating a shift to ePILs. Dynamic capabilities—sensing, seizing, transforming—are needed for ePIL adoption and may pose cross-country collaboration challenges that need to be overcome to accelerate ePIL implementation and operational maturity [14]. Our findings suggest patients are ready for ePILs, particularly for chronic therapies, but implementation should include maintaining a free-of-charge option to obtain a paper PIL at pharmacies (on-demand print) for those who prefer or require it. Strengthened user-testing safeguards is important to support comprehension, including diverse sampling, independent or third-party testing, and protocols for translated PILs [10]. Similarly, content improvements should be implemented that support informed decisions (e.g., rationale for treatment, duration expectations, whether curative/symptomatic/preventive, and, where feasible, quantitative summaries of benefits alongside risks) [13].

A prior Swedish survey conducted in 2014, which explored patients’ use and perceptions of the paper PIL and attitudes toward a potential transition to ePIL, reported more patients preferred paper PILs (with only 17% favouring ePIL) [15]. Our results suggest a shift towards ePIL preference across most age groups, likely reflecting digital adoption and improved electronic access pathways. Nevertheless, our sample was drawn from patients dispensed Turbuhaler products in community pharmacies via convenience sampling, and non-respondents were older on average than respondents. These factors may limit generalisability to other therapeutic areas, acute treatments, over-the-counter products, or patients with lower digital access. Therefore, our results are most directly generalizable to chronic users collecting prescriptions at Swedish community pharmacies. Further research should include other patient groups, medicines, and settings, and evaluate whether access pathway (e.g., barcode scan vs. Internet search) affects findability and preference.

During the COVID-19 pandemic, exemptions were approved for distributing the influenza vaccine without leaflets to expedite vaccination programmes, with patients accessing a PIL through QR codes. A study conducted in multiple European countries examined the switch to ePILs for vaccines, highlighting their potential to enhance information quality and quantity reaching vaccine recipients, as well as to remove barriers to redistributing vaccines between countries [16].

One study limitation is the modest overall response rate of 12%. Most of the patients (83%) that considered to participate did not complete the survey. Since their reasons for not participating are unknown, determining how this may affect the generalisability of our findings is difficult. Similarly, we used a convenience sample from a selection of pharmacies, which may also affect the external validity of the results. Hence, since this study may not be representative for all patients in Sweden, but rather for chronic users collecting prescriptions at community pharmacies, further studies are needed to confirm whether the preference for ePIL is true for more patient groups. Future studies are also needed to incorporate diversified and independent user-testing, test access pathway effects, and assess comprehension outcomes.

Our findings indicate readiness among patients in Sweden to transition to ePILs, with operational and policy steps to ensure safe, equitable implementation. Coordinated stakeholder efforts are needed to formalise contingency plans for outages/cyberattacks to guarantee continuous patient access and to harmonise acceptance of ePIL across countries that share packaging, avoiding cross-border information asymmetries. These measures link preference signals to practical, patient-centred roll-out and align with the EU’s digital objectives while safeguarding equity and understanding.

## Conclusions

This preference survey study indicates that patients in Sweden appear to be ready to transition to an ePIL format from paper PILs. Effective collaboration among various stakeholders will be essential for a smooth implementation, ensuring patient confidence in accessing and reading an ePIL. Patients have the right to access the most current package leaflet to use their medication safely. Still, before phasing out paper leaflets and replacing them with an ePIL, a secured way of providing paper PILs must be in place for patients who prefer or require them. This will have some implications in practice, for instance for pharmacies in providing the additional service of informing patients about the option of having a paper PIL and providing it to patients with such requirements. However, the benefits that follow from a transition to ePIL, both from information accuracy, environmental, and economical point of views, as outlined in this article, clearly call for the transition to take place.

## Supplementary Information

Below is the link to the electronic supplementary material.

Supplementary Material 1

## Data Availability

Data and code underlying the findings described in this manuscript may be obtained in accordance with AstraZeneca’s data sharing policy described at https://astrazenecagrouptrials.pharmacm.com/ST/Submission/Disclosure.

## References

[1] Swedish Medical Products Agency (MPA). Regulation on labelling and package leaflets for human use (HSLF-FS 2021:96). Published 2021. Accessed November 21, 2025. https://www.lakemedelsverket.se/globalassets/dokument/lagar-och-regler/hslf-fs/hslf-fs-2021-96.pdf

[2] Sveriges Riksdag (Swedish Parliament). The Medicinal Products Act (2015:315). Updated 2023. Accessed October 9, 2024. https://www.riksdagen.se/sv/dokument-och-lagar/dokument/svensk-forfattningssamling/lakemedelslag-2015315_sfs-2015-315/

[3] Swedish Medical Products Agency (MPA). ePI—electronic product information initiative. Updated June 3, 2024. Published June 20, 2022. Accessed October 9, 2024. https://www.lakemedelsverket.se/en/permission-approval-and-control/marketing-authorisation/product-information/epi---electronic-product-information-initiative#hmainbody4

[4] European Commission. Notice to applicants—guideline on the packaging information of medicinal products for human use authorized by the Union. Final Rev. 14.8. Published September 2023. Accessed October 9, 2024. https://ec.europa.eu/health/sites/health/files/files/eudralex/vol-2/2018_packaging_guidelines_en.pdf

[5] European Medicines Agency (EMA). Product-information templates—human. Accessed October 9, 2024. https://www.ema.europa.eu/en/human-regulatory-overview/marketing-authorisation/product-information-requirements/product-information-templates-human

[6] European Commission. Report on current shortcomings in the summary of product characteristics and the package leaflet and how they could be improved to better meet the needs of patients and healthcare professionals. Published 2017. Accessed October 9, 2024. https://eur-lex.europa.eu/legal-content/EN/TXT/PDF/?uri=CELEX:52017DC0135

[7] Pelizzari N. Changing roles of patient information leaflets in the UK: a corpus-assisted discourse analysis. Appl Corpus Linguist. 2025;5(2):100129. 10.1016/j.acorp.2025.100129.

[8] European Federation of Pharmaceutical Industries and Associations (EFPIA). Electronic product information: from principles to actions. Published 2021. Accessed October 9, 2024. https://www.efpia.eu/media/589590/electronic-product-information-from-principles-to-actions.pdf

[9] Skogman-Lindqvist C, Lappato-Reiniluoto O, Sirviö M, et al. Benefits and challenges of electronic package leaflet (ePL): review of ePL pilots in hospital settings in Europe. Eur J Pharm Sci. 2023;191:106605. 10.1016/j.ejps.2023.106605. 37821011 10.1016/j.ejps.2023.106605

[10] Pelizzari N. The challenges for EU user testing policies for patient information leaflets. Int J Environ Res Public Health. 2024;21(10):1301. 10.3390/ijerph21101301. 39457274 10.3390/ijerph21101301 PMC11507276

[11] Statistics Sweden (SCB). ICT usage in households and by individuals. Published 2023. Accessed October 9, 2024. https://www.scb.se/en/finding-statistics/statistics-by-subject-area/research-and-the-digital-society/the-digital-society/ict-usage-in-households-and-by-individuals/pong/statistical-news/ict-usage-in-households-and-by-individuals-2023

[12] Swedish Medical Products Agency (MPA). Importance of being careful when buying prescription medicine online. Published 2023. Accessed October 9, 2024. https://via.tt.se/pressmeddelande/3398614/viktigt-att-vara-noggrann-nar-man-koper-receptbelagd-medicin-pa-natet?publisherId=3235477&lang=sv

[13] Dickinson R, Raynor DK, Knapp P, MacDonald J. How much information about the benefits of medicines is included in patient leaflets in the European Union? A survey. Int J Pharm Pract. 2017;25(2):147–58. 10.1111/ijpp.12285. 27405658 10.1111/ijpp.12285

[14] Srai JS, Kumar M, Duc D, et al. Digital transformation of electronic patient information leaflets (ePIL): the case of multinational pharmaceutical manufacturers. Int J Prod Res. 2025;63(13):4887–907. 10.1080/00207543.2024.2445704.

[15] Hammar T, Nilsson AL, Hovstadius B. Patients’ views on electronic patient information leaflets. Pharm Pract (Granada). 2016;14(2):702. 10.18549/PharmPract.2016.02.702. 27382423 10.18549/PharmPract.2016.02.702 PMC4930857

[16] Bamberger M, De Loof H, Marstboom C, et al. Replacing vaccine paper package inserts: a multi-country questionnaire study on the acceptability of an electronic replacement in different target groups. BMC Public Health. 2022;22:156. 10.1186/s12889-022-12510-8. 35073891 10.1186/s12889-022-12510-8 PMC8785016

