# Supplementary material for: Patient Preferences for Electronic Versus Paper Patient Information Leaflets: A Survey Among Patients in Sweden
Source: Ther Innov Regul Sci. 2026 Feb 7;60(3):872–8. doi: 10.1007/s43441-026-00929-9 (PMC13110224; doi:10.1007/s43441-026-00929-9)
Supplement: Supplementary file 1 — Supplementary Material 1 [file 43441_2026_929_MOESM1_ESM.docx]

**SUPPLEMENTARY MATERIAL**

**​Study online survey**

1. Have you read the product information leaflet electronically via FASS (website or app)?

- Yes
- No *(if patients reported ”No”, the questionnaire was locked and patients were requested to read the ePIL via one of the two access pathways and, thereafter, access the survey again)*

1. Which year were you born?

_________________________

1. Which is your gender?

- Woman
- Man
- Other

1. For how long have you (or your relative/friend) used this product?

- This is the first time
- Less than one year
- 1-3 years
- 4-6 years
- More than 6 years

1. When you get a new pharmaceutical product prescribed to you (or your relative/friend), how often do you then read leaflet?

| Never |  |  |  |  |  | Always |
| --- | --- | --- | --- | --- | --- | --- |
| 1 | 2 | 3 | 4 | 5 | 6 | 7 |
|  |  |  |  |  |  |  |

1. If it was possible to choose, would you prefer to receive the leaflet only in paper or only in digital format?

| Only  paper |  |  | Format doesn’t matter |  |  | Only  digital |
| --- | --- | --- | --- | --- | --- | --- |
| 1 | 2 | 3 | 4 | 5 | 6 | 7 |
|  |  |  |  |  |  |  |

1. How was your experience of finding the electronic leaflet?

| Very  easy |  |  |  |  |  | Very difficult |
| --- | --- | --- | --- | --- | --- | --- |
| 1 | 2 | 3 | 4 | 5 | 6 | 7 |
|  |  |  |  |  |  |  |

1. How was your experience of finding the information you searched for in the electronic leaflet?

| Very  easy |  |  |  |  |  | Very difficult |
| --- | --- | --- | --- | --- | --- | --- |
| 1 | 2 | 3 | 4 | 5 | 6 | 7 |
|  |  |  |  |  |  |  |

1. How was your experience of finding the information you searched for in the paper leaflet?

| Very  easy |  |  |  |  |  | Very difficult |
| --- | --- | --- | --- | --- | --- | --- |
| 1 | 2 | 3 | 4 | 5 | 6 | 7 |
|  |  |  |  |  |  |  |

1. Do you normally search for information digitally (e.g., opening hours, directions)?

| Never |  |  |  |  |  | Always |
| --- | --- | --- | --- | --- | --- | --- |
| 1 | 2 | 3 | 4 | 5 | 6 | 7 |
|  |  |  |  |  |  |  |
